# Individual, collective, and transgenerational traumatization in the Yazidi

**DOI:** 10.1186/s12916-017-0965-7

**Published:** 2017-12-11

**Authors:** Jan Ilhan Kizilhan, Michael Noll-Hussong

**Affiliations:** 1 Duale Hochschule Baden-Württemberg Villingen-Schwenningen, Friedrich-Ebert-Strasse 30, D-78054 Villingen-Schwenningen, Germany; 2 Department of Psychosomatic Medicine and Psychotherapy, University of Ulm, Albert-Einstein-Allee 23, D-89081 Ulm, Germany

**Keywords:** Yazidi, Post-traumatic stress disorder, Abuse history, Mental health, Somatic symptom disorder, Transcultural psychiatry, Non-communicable diseases, Migrants, Refugees, Terrorism

## Abstract

In recent years, Islamic terrorism has manifested itself with an unexpectedly destructive force. Despite the fact that Islamic terrorism commences locally in most cases, it has spread its terror worldwide. In August 2014, when troops of the self-proclaimed ‘Islamic State’ conquered areas of northern Iraq, they turned on the long-established religious minorities in the area with tremendous brutality, especially towards the Yazidis. Vast numbers of men were executed, and women and children were abducted and willfully subjected to sexual violence. With the aim of systematic destruction of the Yazidi community, the religious minority was to be eliminated and the will of the victims broken. The medical and mental health issues arising from the combination of subjective, collective, and cultural traumatization, as well as the subsequent migrant and refugee crisis, are therefore extraordinary and require novel and wise concepts of integrated medical care.

## Background

The Yazidis are a Kurdish minority group, distinguished in terms of religion rather than through ethnic or linguistic differences. The majority of Kurds were forced to convert to Islam [1]; however, the Yazidis, who resisted this, regard themselves as followers of the oldest religion in the world. Yazidis live predominantly in present-day northern Iraq, with their total population worldwide being estimated at 800,000 to 1,000,000 [2].

The Islamization of the Kurdish areas in Iraq, Iran, Syria, and Turkey brought with it an odyssey of persecution; it is estimated that 74 genocides against the Yazidis have been carried out in the past 800 years [3]. Since the attack by the so called ‘Islamic State’ (IS) at the beginning of August 2014, more than 7000 Yazidis have been killed and thousands of families have been held hostage in their villages and murdered if they refused conversion to Islam, over 5800 young girls have been abducted, raped, sold in Arab markets, enslaved, and killed, and more than 20,000 Yazidis have fled to Turkey and approximately 400,000 to the Kurdish region [4].

## From children at war towards war-damaged patients

From the beginning, IS deliberately targeted boys aged between 8 and 14 years old, tearing them away from their families and training them as child soldiers [5] through daily religious indoctrination, martial arts, and resilience against pain and brutality. Girls from the age of 8 are raped in order to impregnate them; some of these young Yazidis have managed to flee captivity or have been freed in return for a ransom payment. These severely traumatized young women now live in approximately 24 refugee camps near Duhok and Zakho in northern Iraq, each housing up to 28,000 refugees. Treatment options within these camps are severely limited or non-existent. Tragically, some of these girls kill themselves in order not to live with the supposed ‘dishonor’ [6], which results from the fact that Yazidis normally live according to a caste system that does not allow any sexual contact or marriage outside the caste or with non-Yazidis. Traditionally, Yazidis entering a relationship with non-Yazidis are ostracized from the community [2].

## Collective trauma and traumatization across generations

In addition to representing a source of traumatization by itself, the genocide perpetrated by IS has resurfaced memories from past genocides and massacres of the Yazidis’ ancestors. Thus, current Yazidi victims are (re)experiencing multiple traumatization, as well as feelings of helplessness through repeated Islamic terrorization. Indeed, similar behavior patterns are being observed in Yazidi people to those observed in people who experienced the Holocaust [7], namely feelings of insecurity, tension, worry about their children’s survival, and feelings of powerlessness and helplessness.

From a clinical point of view, the traumatization of women has brought about physical suffering in addition to chronic mental illness [8], including diarrhea, frequent colds, and psychosomatic illnesses such as skin problems, asthma, or pain [9, 10]. Thus, patients often do not understand the specific reason for their condition (e.g., physical pain caused by internal psychological conflicts or traumatization) and cannot allocate it to their understanding of an illness. Some children who survived the genocide complain of headaches, wet themselves regularly, and are jumpy, careful, and distanced when in contact with strangers. Moreover, some of the children are quick to react aggressively, feel insecure, and (will) have major issues (re)integrating into society.

On account of the disastrous situation in Iraq and Syria, the government of Baden-Württemberg state, Germany, decided to receive and treat approximately 1000 Yazidi people in need of protection, in particular young women who have been held captive by IS [11]. At present, 1000 women and children in Baden-Württemberg, 30 in Schleswig-Holstein, and 70 in Lower Saxony are receiving care and support from medical staff, psychologists, and social workers.

## Treatment and care

The treatment regimens for individual, collective, and transgenerational trauma are closely interrelated; indeed, treatment may span generations and requires a culture-sensitive approach [12]. The concept of post-traumatic stress disorder can be applied to all ethnic groups. Yet, the different notions of health and/or illness as well as cultural or medical treatment traditions when dealing with traumatic experiences require different concepts or therapeutic modifications [13–16].

A basic requirement is a secure environment in which the person does not feel the threat of persecution or any other danger. Only this sense of security will allow open dialogue regarding their experiences and acceptance of the treatment and therapists. Even the recognition of cognitions, emotions, the definition of self, individual and collective identity, and the way in which disorders manifest themselves (for example, women increasingly complain of headaches), makes conventional treatment difficult since there is often no concordance with known diagnostic criteria [17]. Women repeatedly report, in great detail, the burden of their ancestors using the word ‘Ferman’ (equivalent to the Holocaust) [18], and are not able to make the link between this and their own traumatization, which can lead to a lack of understanding and impatience among physicians and therapists [19].

A further problem during treatment is trauma confrontation [20, 21]. The Yazidis are familiar with the practice of ‘narration’, freely speaking about disasters, which can be used to good effect as a resource [22]. Nevertheless, traditional expositional therapy with survivors who have completed their socialization in a collective society up to adulthood is not always effective [23–25], and may even be counterproductive, reducing compliance and increasing the drop-out rate. Against this background, it remains an open question whether suppression and avoidance might be better coping strategies. In many cultures, this represents a successful coping mechanism, and this is particularly true in collectivist societies in which social harmony is the highest priority [26]. Particularly in this case, the healing process is determined by the cultural and social context, and all efforts are made to ensure that the victim does not ‘lose face’. When correctly applied (e.g., by means of social support, social contact, and when embedded in social structures), narration can be accepted by the victim and can be experienced as a healing process. In traditional societies, the telling of stories (narration) is a trusted psychological tool; in fact, this tool was still known to the Mayas [27].

The collective experiences of terror and abuse, part of the collective memory of the Yazidis, difficult as they may be, may (from a psychotherapeutic point of view) help a Yazidi to come to terms with their individual trauma by strengthening their resilience. Resilience strengthening is of great significance for the treatment of terror survivors.

## Conclusions

Thus, in order to overcome trauma, stability, security, a sense of orientation, self-esteem, and intimacy are needed. Irrespective of cultural aspects and an ethnic sense of belonging, it is crucial that both therapists and patients understand the multifaceted illness and are able to categorize it correctly. The therapist must adapt their explanations to the level of the patient’s educational and cultural background. A different understanding of health and illness, of traditional medical care in the home country, and of the role of the individual and collective can all play a great part in the diagnostics and treatment of people from various cultural backgrounds with physical reactions to extreme stress.

To what extent psychotherapeutic trauma management is possible also depends on the manner in which societies deal with the topic of sexuality, violence, and transgenerational stress. High moral conceptions, limitations, and internalized attitudes to ‘honor’ and the ‘violation of honor’ can lead to considerable worry and the fear of collective exclusion. In this respect, feelings of shame play a particular role. In a ‘shame culture’, it is not so much the incident itself and the perpetration of a possible violation of the norm that plays a part, but rather how one can ‘save face’ in front of others. Alternative approaches to treatment that have an interdisciplinary and culture-sensitive focus, in which psychiatrists, psychotherapists, and physicians co-operate with other professionals with sufficient knowledge of the patients’ cultural imprint and who take this into account, are particularly important.
